# Computational reconstruction of transcriptional regulatory modules of the yeast cell cycle

**DOI:** 10.1186/1471-2105-7-421

**Published:** 2006-09-29

**Authors:** Wei-Sheng Wu, Wen-Hsiung Li, Bor-Sen Chen

**Affiliations:** 1 Lab of Control and Systems Biology, Department of Electrical Engineering, National Tsing Hua University, Hsinchu, 300, Taiwan; 2 Department of Evolution and Ecology, University of Chicago, 1101 East 57th Street, Chicago, IL, 60637, USA; 3 Genomics Research Center, Academia Sinica, Taipei, Taiwan

## Abstract

**Background:**

A transcriptional regulatory module (TRM) is a set of genes that is regulated by a common set of transcription factors (TFs). By organizing the genome into TRMs, a living cell can coordinate the activities of many genes and carry out complex functions. Therefore, identifying TRMs is helpful for understanding gene regulation.

**Results:**

Integrating gene expression and ChIP-chip data, we develop a method, called MOdule Finding Algorithm (MOFA), for reconstructing TRMs of the yeast cell cycle. MOFA identified 87 TRMs, which together contain 336 distinct genes regulated by 40 TFs. Using various kinds of data, we validated the biological relevance of the identified TRMs. Our analysis shows that different combinations of a fairly small number of TFs are responsible for regulating a large number of genes involved in different cell cycle phases and that there may exist crosstalk between the cell cycle and other cellular processes. MOFA is capable of finding many novel TF-target gene relationships and can determine whether a TF is an activator or/and a repressor. Finally, MOFA refines some clusters proposed by previous studies and provides a better understanding of how the complex expression program of the cell cycle is regulated.

**Conclusion:**

MOFA was developed to reconstruct TRMs of the yeast cell cycle. Many of these TRMs are in agreement with previous studies. Further, MOFA inferred many interesting modules and novel TF combinations. We believe that computational analysis of multiple types of data will be a powerful approach to studying complex biological systems when more and more genomic resources such as genome-wide protein activity data and protein-protein interaction data become available.

## Background

A transcriptional regulatory module (TRM) is a set of genes that is regulated by a common set of TFs. By organizing the genome into TRMs, a living cell can coordinate the activities of many genes and carry out complex functions. Therefore, identifying TRMs is useful for understanding cellular responses to internal and external signals. The advances of high-throughput genomic tools such as DNA microarray [1,2] and chromatin immunoprecipitation-DNA chip (ChIP-chip) [3,4] have made the computational reconstruction of TRMs of a eukaryotic cell possible.

Genome-wide gene expression analysis has been used to investigate TRMs controlling a variety of cellular processes in yeast [5-9]. Clustering and motif-discovering algorithms have been applied to gene expression data to find sets of co-regulated genes and have identified plausible binding motifs of their TFs [7,10,11]. Such approaches have also been expanded to incorporate previous knowledge about the genes, such as cellular functions [12] or promoter sequence motifs [13]. Moreover, some researchers used model-based approaches such as random Boolean networks [14] and Bayesian networks [15,16] to infer regulatory network architectures. However, this approach provides only indirect evidence of genetic regulatory interactions and does not identify the relevant TFs. On the other hand, the ChIP-chip technique was developed to identify physical interactions between TFs and DNA regions. Using ChIP-chip data, Simon *et al. *[17] investigated how the yeast cell-cycle gene-expression program is regulated by each of the nine major transcriptional activators. Lee *et al. *[18] constructed a network of TF-gene interactions and Harbison *et al. *[19] constructed an initial map of yeast's transcriptional regulatory code. However, ChIP-chip data alone cannot tell whether a TF is an activator or a repressor and, most importantly, ChIP-chip data are noisy and, depending on the chosen *p*-value cutoff, include many false positive or false negative TF-DNA binding relationships.

Since gene expression and ChIP-chip data provide complementary information, some researchers [20-22] have integrated both types of data in their studies. However, most previous studies except the GRAM algorithm [21] assumed that a gene is regulated by a TF only if the *p*-value of TF-gene binding in the ChIP-chip data is ≤ 0.001, thus suffering a false negative rate of ~24% in determining TF-gene binding [19].

In order to reduce the high false negative rate, we develop a method, called Temporal Relationship Identification Algorithm (TRIA), that uses the information provided by gene expression data to alleviate the effect of using a stringent threshold in determining TF-gene binding. A TF-gene pair is said to have a positively (negatively) temporal relationship if the gene's expression profile is positively (negatively) correlated with the TF's regulatory profile possibly with time lags (see Methods). TRIA identifies TF-gene pairs with a temporal relationship. We define that a TF binds to a specific gene if (1) the *p*-value for the TF to bind the gene is ≤ 0.001 in the ChIP-chip data or (2) 0.001 <*p *≤ 0.01 and the TF-gene pair has a temporal relationship. That is, we allow the *p*-value cutoff to be relaxed to 0.01 if the TF-gene pair has a temporal relationship. Our approach is different from the GRAM algorithm [21], which relied on sets of co-expressed gene to relax the stringent *p*-value cutoff.

From the above procedure, we derive a binding score matrix. Then we develop the MOdule Finding Algorithm (MOFA) that combines this binding score matrix with the gene expression matrix to reconstruct TRMs of the yeast cell cycle (see Methods). For each of the five cell cycle phases (M/G1, G1, S, S/G2 and G2/M), MOFA exhaustively searches for all possible TF combinations and find their target genes. Once the set of target genes to which a common set of TFs bind is inferred, MOFA identifies a subset of these target genes whose gene expression profiles are positively correlated possibly with time lags. That is, the genes of a module not only share a common set of TFs but also have positively (time-shifted) correlated expression profiles. Our gene module is more general than that of GRAM algorithm [21], which only searched co-expressed genes to form a module. MOFA reconstructs 87 TRMs. We then validate the biological relevance of each inferred TRM using existing experimental data, enrichment for genes in the same MIPS functional category [23], known DNA-binding motifs [7], etc.

## Results

**Figure 1:**
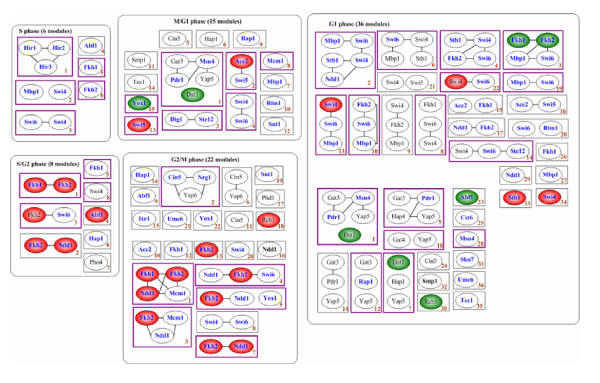
**The 87 TRMs identified in this study**. Each rectangle stands for a module and the ovals in a rectangle indicate the TFs that regulate the module. TF name is colored blue if its function is consistent with one of the module's over-represented MIPS functional categories with adjusted *p*-value < 0.05 (after the Bonferroni correction for multiple tests) using the cumulative hypergeometric distribution or black if not. Two ovals are connected by a line if previous studies indicated that the two TFs interact with each other physically (forming a protein complex), functionally (in the same cellular process) or statistically (co-occurrence) [19-22,24]. An oval is colored red (green) if the TF is identified as an activator (repressor). The periphery of a rectangle is colored purple if this module implicates important TF combinations or is discussed in the text.

By integrating the gene expression and ChIP-chip data, MOFA identified 87 TRMs, which together contain 336 distinct genes regulated by 40 distinct TFs (see Figure 1 and Additional file 1). In the literature [7,23-25], 139 of the 336 genes and 30 of the 40 TFs are known to be involved in the cell cycle.

### Validation of the identified modules

Analysis of the identified modules suggests that MOFA identifies biologically relevant groups of genes. First, 83 of the 87 modules contain genes that are known to be involved in the cell cycle (see Additional file 1). Second, 51% (44/87) identified module includes groups of genes that function in the same cellular process: each of these modules contains at least one over-represented MIPS functional category with adjusted *p*-value < 0.05 (after the Bonferroni correction for multiple tests) using the cumulative hypergeometric distribution (see Additional file 2). Third, the modules are generally accurate in assigning TFs to sets of genes whose functions are consistent with the TFs' known roles. We found that the regulatory functions of the 71% (120/169 counting multiplicity) TFs are consistent with one of their modules' over-represented MIPS functional categories with adjusted *p*-value < 0.05 (see Figure 1). As an example, Dig1 and Ste12 are known to regulate mating and pseudohyphal growth [26] and M/G1 is the critical phase for these processes. All five genes (*FUS1*, *GPA1*, *KAR4*, *SST2*, *TEC1*) of the {Ste12, Dig1} module are important for mating, pseudophyphal growth, or pheromone response. Fourth, 33% (188/568 counting multiplicity) genes are known by previous studies to be regulated by at least one of the TFs that we assigned to the module (see Additional file 1). Fifth, the genes of a module usually have the same binding motifs of the important cell cycle TFs such as SCB (bound by SBF), MCB (bound by MBF), SFF (bound by SFF), ECB (bound by Mcm1) and SWI5 (bound by Ace2 and Swi5). We found that in the majority of cases (36/45) in which a module is controlled by at least one of the important cell cycle TFs (SBF, MBF, SFF, Mcm1, Ace2 and Swi5), there always exist genes that have the known binding motifs of the corresponding TFs (see Additional file 1). Finally, in most cases in which a module is controlled by more than one TF, there is evidence that these TFs may interact physically or functionally (see Figure 1). About 59% (70/118) of the TF interactions that we identified have been experimentally proven or identified by computational algorithms [19-22,24]. Taken together, these results provide evidence that MOFA identifies not only sets of biologically related genes, but also TFs that individually or cooperatively control these genes.

### Identification of important cell cycle TFs and their combinations

**Figure 2:**
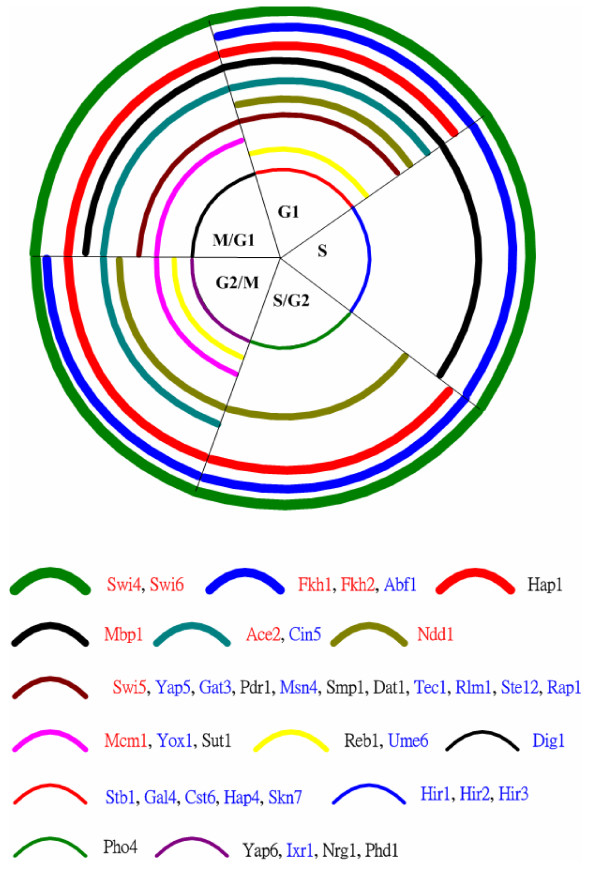
**The cell cycle phases in which each of the 40 identified TFs carries out its regulatory function**. Nine well-known cell cycle TFs are colored red and another 21 TFs that are also involved in the cell cycle [23-25] are colored blue.

MOFA identified 40 TFs that regulate genes of the yeast cell cycle and Figure 2 shows the cell cycle phases in which these TFs carry out their regulatory functions. Table 1 lists these 40 TFs according to the number of target genes. The nine well-known cell cycle TFs (Ace2, Fkh1, Fkh2, Mbp1, Mcm1, Ndd1, Swi4, Swi5, and Swi6) are ranked within the top 14, suggesting the effectiveness of MOFA to find important cell cycle TFs. Moreover, we found another 21 TFs (Abf1, Cin5, Cst6, Dig1, Gal4, Gat3, Hap4, Hir1, Hir2, Hir3, Ixr1, Msn4, Rap1, Rlm1, Skn7, Stb1, Ste12, Tec1, Ume6, Yap5, and Yox1) that are relative to the cell cycle process, consistent with the previous studies [23-25]. The remaining 10 TFs (Dat1, Hap1, Nrg1, Pdr1, Phd1, Pho4, Reb1, Smp1, Sut1, and Yap6) are putative cell cycle related TFs. Among them, Hap1 is more plausible than the others to be related to the cell cycle process since the number of cell cycle genes that it regulates is much larger than that of the others (see Table 1). Actually, it has been shown that Hap1 (also called Ape1 AP endonuclease) regulates *APE1 *[27]. Ape1 is a dual function enzyme and its cell cycle-dependent expression might affect both DNA repair and the activity of various transcription factors as a function of the cell cycle [27]. This evidence validates that MOFA has the ability to find novel TFs which may play a role in the cell cycle or are involved in other cellular processes that have crosstalk with the cell cycle process.

**Table 1:** The number of genes regulated by each of the 40 TFs in each cell cycle phase.

TF	All phases	M/G1	G1	S	S/G2	G2/M
**Swi4**	78	5	45	9	9	10
**Swi6**	74	5	50	7	5	7
**Fkh2**	67		14	5	15	33
**Mbp1**	57	5	47	5		
**Fkh1**	53		17	5	18	13
**Ndd1**	50		12		5	33
*Abf1*	34		10	5	10	9
**Swi5**	34	24	10			
*Cin5*	33	8	7			18
Hap1	30	9	5		5	11
*Stb1*	24		24			
**Mcm1**	22	7				15
*Yap5*	22	5	17			
**Ace2**	21	6	10			5
*Gat3*	20	5	15			
Pdr1	17	5	12			
*Yox1*	16	5				11
*Msn4*	15	5	10			
Reb1	15		8			7
Smp1	15	8	7			
Dat1	13	5	8			
Sut1	13	5				8
*Tec1*	13	5	8			
*Rlm1*	12	7	5			
*Ste12*	12	5	7			
*Ume6*	11		6			5
Yap6	11					11
*Rap1*	10	5	5			
*Gal4*	6		6			
*Hir1*	6			6		
*Hir2*	6			6		
*Hir3*	6			6		
*Cst6*	5		5			
*Dig1*	5	5				
*Hap4*	5		5			
*Ixr1*	5					5
Nrg1	5					5
Phd1	5					5
Pho4	5				5	
*Skn7*	5		5			

Nine well-known cell cycle TFs are bold-faced and another 21 TFs that are also involved in the cell cycle [23-25] are in italic type. The TFs are ordered by the number of their target genes in all phases.

TF combinations and their target genes that are important for each cell cycle phase are also found. We found that different combinations of a fairly small number of TFs are responsible for regulating a large number of genes in different cell cycle phases. Detailed discussions of the TF combinations and their target genes in each specific cell cycle phase are given below.

### The M/G1 phase

Ace2 and Swi5 have been shown to control certain genes expressed in M/G1 [28]. We successfully found that {Ace2, Swi5} and {Swi5} regulate, respectively, modules 2 and 13 in M/G1. Both Ace2 and Swi5 were found to regulate *EGT2*, whose product is involved in cell wall biogenesis and cytokinesis. Swi5 also regulates *PCL9*, whose product is the only cyclin known to act in M/G1 [29] and *SIC1*, whose product is a cyclin regulator that inhibits the Cdc28-Clb kinase activity. Furthermore, Swi5 regulates several Y' genes, which are a subgroup of a larger group of sub-telomeric genes that share DNA sequence similarity and whose expression peaks in early G1 [7].

It is known that in the absence of Ndd1 and Fkh2, Mcm1 participates in the regulation of genes essential for cellular functions specific to late mitosis and early G1 [30,31]. Indeed, we found that {Mcm1} regulates module 8 in M/G1. It regulates *CDC46*, which encodes a protein involved in pre-replication complex formation and *AGA2*, which involves in mating. In addition, Yox1 was recently characterized as a binding partner of Mcm1 in M/G1 [30]. We found that {Yox1}, acting as a repressor, regulates module 15 in M/G1. Three genes *CDC46*, *PIG1 *and *YOR066W *are found to be regulated by both Mcm1 and Yox1, confirming that Yox1 and Mcm1 may co-regulate a group of genes.

In addition, some cell-wall genes are known to be under the control of the M-phase regulator Mcm1 or the G1-phase regulator SBF. The M/G1 phase is a crucial time for cell wall synthesis because the bud separates from the mother right after the M/G1 phase. We successfully found TF combinations {Mcm1} and {Swi4, Swi6}, whose common target genes include *SWI4*, which encodes a late G1 TF, and *UTR2*, which is involved in cell-wall organization and polarized growth. The dual regulation of *SWI4 *by Mcm1 and Swi4 has been shown previously [31].

We identified {Dig1, Ste12} to regulate module 3 in M/G1. The genes of this module include *FUS1*, *GPA1*, *KAR4*, *SST2*, and *TEC1*, which are important for mating or pseudohyphal growth. Dig1 and Ste12 are known to regulate mating and pseudohyphal growth [26], supporting the biological relevance of our finding. We also found novel TF combinations. For example, {Dat1, Gat3, Msn4, Pdr1, Yap5} is identified to regulate a group of genes that are similar to sub-telomerically encoded proteins.

### The G1 phase

Previous molecular and genetic analysis suggested that SBF and MBF are important activators of genes essential for cellular functions specific to late G1 [17,32]. Our result confirms this model: 10 out of the 36 modules in G1 are regulated by MBF or SBF. SBF regulates *BUD9*, *EXG1 *(both of module 8), *GAS1*, *MNN1*, *OCH1 *and *PSA1 *(all of module 22). These genes are involved in the morphological changes associated with cell budding. MBF controls *PDS5*, *RAD51*, *RNR1 *(all of module 3), *DUN1*, *IRR1 *and *RAD27 *(all of module 19). These genes are involved in DNA replication and repair. Moreover, the targets of SBF and MBF also include key cell cycle regulators. Both SBF and MBF were found to regulate *CLN1*, *CLB6 *(both of module 2) and *PCL1 *(of module 6). In addition, SBF regulates *PCL2 *(of module 14) and MBF regulates *CLB6 *(of module 2).

We found that Stb1 together with SBF (Swi4+Swi6) or MBF (Mbp1+Swi6) regulates modules 2, 4 and 6 in G1. It has been known that Stb1 binds to Swi6 *in vitro *and is thought to interact with Swi6, a subunit of both SBF and MBF, to regulate transcription *in vivo *[33]. Also, consistent with our results, Kato *et al. *[22] claimed the presence of the complexes Stb1+Swi6+Swi4 and Stb1+Swi6+Mbp1. Moreover, we found that {Ste12, Swi4, Swi6} regulates module 14 in G1, which is also consistent with the result of [22].

We found that Fkh1/Fkh2 combines with MBF/SBF to regulate modules 3, 4, 8 and 10 in G1. It is known that Fkh1 and Fkh2 regulate genes expressed in G2/M and also genes expressed in other cell cycle phases [17], supporting our result. We also found some novel TF combinations. For example, {Dat1, Gat3, Msn4, Pdr1, Yap5}, which is also found in M/G1, {Gat3, Hap4, Pdr1, Yap5}, {Dat1, Hap1, Yap5}, {Gat3, Rap1, Yap5}, {Gal4, Yap5} and {Msn4} are all identified to regulate genes whose products are similar to sub-telomerically encoded proteins. All these genes share DNA sequence similarity and are found in Y' elements, which are located at chromosomes ends [7].

### The S phase

We found that {Fkh2} regulates various genes that encode proteins associated with chromatin structure including histone genes *HHF1 *and *HHT1 *(both of module 6). We found that {Fkh1} regulates *TEL2 *(of module 5), a telomere length regulator, and *ARP7 *(of module 5), a subunit of the chromatin remodeling Swi/Snf complex. Histone genes can be found in the {Fkh1}, {Fkh2}, {Swi4, Swi6} and {Mbp1, Swi4} modules, suggesting that SBF, Fkh1 and Fkh2 probably regulate histone genes. Our result is consistent with a few genomic studies [18,34] that indicated the involvement of SBF and Fkh1/Fkh2 in regulating S phase genes. In addition, we successfully identified {Hir1, Hir2, Hir3} to regulate six histone genes (*HTA1*, *HTB1*, *HHT1*, *HHF1*, *HHT2*, *HHF2*) of module 1 in the S phase, supported by existing experimental results [35]. In summary, we suggest that SBF and Fkh1/Fkh2 are activators and Hir1, Hir2 and Hir3 are repressors of histone genes.

### The S/G2 and G2/M phases

Simon *et al. *[17] and Lee *et al. *[18] indicated the involvement of SBF and Fkh1/Fkh2 in regulating S/G2 genes. We confirmed that Fkh1, Fkh2, Swi4 and Swi6 are important TFs in this phase since five out of the eight modules in S/G2 are regulated by at least one of these TFs. Fkh2, Swi4 and Swi6 are identified to regulate *SIM1*, which is involved in cell cycle control, and Fkh1 is identified to regulate *CLB4*, which encodes an S/G2 cyclin.

Previous studies have demonstrated that Mcm1 collaborates with Ndd1 and Fkh1/Fkh2 to regulate genes necessary for both entry into and exit from mitosis [36,37]. We successfully identified this TF combination to regulate module 1 in G2/M. Four of the seven genes identified in this module have an SFF (bound by Ndd1+Fkh1/Fkh2) or ECB (bound by Mcm1) motif (see Additional file 1). The Mcm1+Ndd1+Fkh1/Fkh2 protein complex regulates transcription of *CLB2 *(of module 1), whose product is necessary to enter mitosis. Furthermore, SBF and MBF regulate *SWE1 *(of module 13 in G1) and *GIN4 *(of module 13 in G1). Swe1 is a protein kinase that regulates the G2/M transition by inhibition of Cdc28-Clb2 kinase activity and Gin4 regulates Swe1 [38]. The Mcm1+Ndd1+Fkh1/Fkh2 protein complex also sets the stage for exit from mitosis at several levels [17]. First, they regulate two key M/G1 TFs: *SWI5 *(of module 3) and *ACE2 *(of module 1). Second, they regulate *CDC20 *(of module 1), an activator of the anaphase promoting complex (APC). Finally, these activators regulate *SPO12 *(of module 3), which encodes a protein that regulates the mitotic exit.

It has been suggested that Fkh2 has a more prominent role than Fkh1 in G2/M transcription [36]. Our analysis agrees with this suggestion since the number of G2/M genes regulated by Fkh2 is much larger than that of Fkh1 (see Table 1). We also found novel TF combinations. For example, we found that SFF instead of combining with MCM1 can also combine with Swi6 or Yox1 to regulate G2/M genes and {Cin5, Nrg1, Yap6} is identified to regulate a group of genes with unknown functions.

## Discussion

### Relationships between two TFs of a module

The relationships between two TFs that regulate the same module fall into three categories. First, both TFs bind DNA in the same promoter region but do not interact with each other. Different TFs may regulate the target gene to execute different functions in different cellular processes. Indeed, we found that TFs in this category usually regulate genes that are required for multiple cellular processes. For example, we found that {Ste12, Swi4, Swi6} regulates module 14 in G1. Since Ste12 and SBF (Swi4+Swi6) are both DNA-binding TFs and there is no evidence that Ste12 interacts with SBF, the relationship between Ste12 and SBF belongs to this category. Ste12 is a regulator of the mating or pseudohyphal growth pathway and SBF is an important regulator in the G1 phase. This indicates that there may exist crosstalk between these two cellular processes. That is, the TF combination {Ste12, Swi4, Swi6} probably regulates genes needed for the G1 phase and also independently needed for mating, confirming the results of [22]. Second, both TFs bind DNA and interact with each other. For example, we found that {Fkh2, Ndd1, Mcm1} regulates module 3 in G2/M. Both Mcm1 and Fkh2 bind DNA and these two proteins together recruit Ndd1 to form a protein complex to control the transcription of G2/M genes [36]. Third, only one TF binds DNA and the other TF regulates the target genes through binding to the DNA-binding TF. For example, {Mbp1, Swi6} and {Swi4, Swi6} are found to regulate, respectively, modules 19 and 22 in G1. MBF (Mbp1+Swi6) functions in DNA replication, and SBF (Swi4+Swi6) predominantly controls the expression of budding and cell-wall genes [4]. Since Swi6 is a non-DNA-binding cofactor of Swi4 and Mbp1, the relationship between Swi6 and Swi4/Mbp1 falls into the third category.

### Advantages of MOFA

MOFA has two features that make it more powerful than current methods. First, it can reduce false negatives in determining binding events in the ChIP-chip data. Most researchers except for Bar-Joseph *et al. *[21] have chosen a relatively stringent *p*-value threshold (0.001) to determine binding in order to reduce false positives at the expense of false negatives [18-20,22]. In comparison, MOFA allows the *p*-value cutoff to be relaxed to 0.01 if a TF-gene pair has a temporal relationship. (Our approach is different from the GRAM algorithm [21], which relied on sets of co-expressed gene to relax the stringent *p*-value cutoff.) As an example, consider Swi5, a well-characterized cell cycle TF in M/G1. The {Swi5} module we inferred contains 18 genes that have similar expression patterns (see Additional file 4). Four of these genes (*YOR264W*, *PST1*, *SIC1 *and *YHB1*) would not have been identified as Swi5 targets using the stringent *p*-value threshold (0.001). Previous studies identified these four genes as true targets of Swi5 [7,18]. This attests to the ability of MOFA to lower the rate of false negatives without substantially increasing the rate of false positives. Overall, 87 of the 988 unique TF-gene interactions discovered by MOFA would not have been detected using the current ChIP-chip data with the stringent *p*-value cutoff (0.001). In addition, 312 of the 988 unique TF-gene interactions are supported by gene expression data. That is, each of the 312 TF-gene pairs is identified to have a temporal relationship (see Additional file 1).

Second, MOFA can determine the role of a TF in regulating genes of a module. A TF is said to be an activator (repressor) of a module if the *p*-value of observing TF-gene pairs of the module having a positively (negatively) temporal relationship is ≤ 0.001. The *p*-value is the probability that an observation would be made by chance, and is calculated using the cumulative binomial distribution [39]. We found nine activators (Abf1, Ace2, Fkh1, Fkh2, Ndd1, Reb1, Stb1, Swi4 and Swi5) and six repressors (Abf1, Dat1, Fkh1, Fkh2, Reb1 and Yox1), consistent with the results of previous studies [40-52]. Interestingly, four TFs (Abf1, Fkh1, Fkh2 and Reb1) are capable of being activators and repressors to regulate different modules. Table 2 provides the detailed discussion of how we assign the regulatory roles of TFs and the known experimental evidence that supports our findings.

**Table 2:** Identifying regulatory roles of TFs. MOFA can determine the regulatory role of a TF in regulating genes of a module.

TF	Phase (Module Number)	Regulatory Role	*P*-value	Evidence from Literature
Abf1	S/G2 (4)	Activator	6 × 10^-5^	[40]
Abf1	G1 (23)	Repressor	0.001	[41]
Fkh1	S/G2 (1); G2/M (1)	Activator	3 × 10^-8^; 1 × 10^-7^	[37]
Fkh1	G1 (3)	Repressor	3 × 10^-5^	[42]
Fkh2	S/G2 (1) (2) (3) ; G2/M (1) (3) (4) (5) (7) (13)	Activator	3 × 10^-8^; 3 × 10^-7^; 3 × 10^-5^; 1 × 10^-7^; 6 × 10^-9^; 3 × 10^-5^; 9 × 10^-5^; 6 × 10^-9^; 3 × 10^-5^	[37]
Fkh2	G1 (3)	Repressor	3 × 10^-5^	[42]
Reb1	G2/M (18)	Activator	2 × 10^-4^	[43]
Reb1	G1 (30)	Repressor	2 × 10^-5^	[44-46]
Ace2	M/G1 (2)	Activator	9 × 10^-5^	[28,47]
Ndd1	S/G2 (2); G2/M (1) (7)	Activator	3 × 10^-5^; 1 × 10^-7^; 6 × 10^-6^	[48,49]
Stb1	G1 (33)	Activator	2 × 10^-11^	[33,50]
Swi4	G1 (13) (22) (34)	Activator	6 × 10^-9^; 1 × 10^-6^; 2 × 10^-4^	[51]
Swi5	M/G1 (13)	Activator	6 × 10^-8^	[28,47]
Dat1	M/G1 (1); G1 (1) (7)	Repressor	3 × 10^-5^; 3 × 10^-7^; 3 × 10^-5^	[52]
Yox1	M/G1 (15)	Repressor	3 × 10^-7^	[30]

A TF is said to be an activator/repressor of a module if the P-value of observing TF-gene pairs of the module having positively/negatively (time-shifted) correlated profiles is ≤ 0.001. The P-value is the probability that an observation would be made by chance, and is calculated using the cumulative binomial distribution [39]: $P(x\geq n_{0})=\sum_{x=n_{0}}^{N}\left[\frac{N!}{x!(N-x)!}\right]p^{x}{\left(1-p\right)}^{N-x}$ where N is the total number of genes in a module, n0 is the number of genes that have temporal relationships with the TF, and p is the probability of observing an arbitrary gene in the genome that has a temporal relationship with the TF.

MOFA is more powerful than GRAM algorithm [21] in two ways. First, MOFA has the ability to assign a TF to be an activator or/and a repressor (see Table 2). On the contrary, GRAM algorithm cannot find any repressors or activators that are correlated with its target genes with time lags since GRAM algorithm regards a TF to be an activator only when the expression profiles of the TF and the genes in the corresponding module are co-expressed. For example, GRAM algorithm found only two (Fkh1 and Fkh2) of the nine activators and none of the six repressors that are found by MOFA (see Table 2). Second, MOFA is more powerful than GRAM algorithm to find out co-regulated genes that are not co-expressed. While GRAM algorithm assumed that the genes of a module are co-expressed, MOFA allows the genes of a module to be positively correlated with time lags. Since it is known that co-regulated genes may not be co-expressed [53,54], the relaxation of co-expressed assumption of GRAM algorithm makes MOFA have a better ability to reconstruct gene modules with biological relevance. For example, MOFA identified four genes (*YOR264W*, *PST1*, *SIC1 *and *YHB1*) as Swi5 targets ({Swi5} module in M/G1) which is supported by previous studies [7,18]. However, none of them was found by GRAM algorithm.

### Parameter settings of MOFA

The choices of both the relaxed *p*-value and time-lag parameter have biological meanings. Two previous papers [18,19] used a statistical error model to assign a *p*-value of the binding relationship of a TF-gene pair. They found that if *p *≤ 0.001, the binding relationship of a TF-gene pair is of high confidence and can usually be confirmed by gene-specific PCR. If *p *> 0.01, the binding relationship of a TF-gene pair is of low confidence and cannot be confirmed by gene-specific PCR most of the time. However, if 0.001 <*p *≤ 0.01, the binding relationship of a TF-gene pair is ambiguous and can be confirmed by gene-specific PCR in some cases but not in the other cases. Our aim is to solve this ambiguity. This is why we choose 0.01 to be the relaxed *p*-value. We say that an ambiguous binding relationship of a TF-gene pair is plausible if 0.001 <*p *< 0.01 and if this TF-gene pair has a temporal relationship. As to the time-lag parameter, its value is chosen to make the maximal time lag approximately equal to two consecutive cell cycle phases because Simon *et al. *[17] found cases where a cell cycle TF that expresses in one phase of the cell cycle can regulate genes that function in the next phase.

Increasing the value of the relaxed *p*-value or the time-lag parameter may introduce some false positive binding relationships of TF-gene pairs into the binding score matrix. On the other hand, decreasing the value of the relaxed *p*-value or the time-lag parameter may fail to rescue some false negative binding relationships of TF-gene pairs. A binding score matrix is used to construct an original TRM and MOFA refines the TRM by identifying a subset of these co-regulated genes in a TRM whose gene expression profiles are highly positively correlated possibly with time lags. MOFA can filter out false positives to some extent because the expression profiles of false positives are unlikely by chance to be similar to those of the highly positively time-delayed correlated genes identified by MOFA. As to the false negative problem, MOFA cannot alleviate the harmful effect since these plausible binding relationships of TF-genes pairs are not included in the first place. That is, false negative problem is a more serious issue than the false positive problem in MOFA. Therefore, if users have no idea about the appropriate values of the relaxed *p*-value and the time-lag parameter, they should first try larger values since MOFA has the ability to reduce this kind of noises.

### Refining clusters from Spellman et al

Spellman *et al. *[7] used a hierarchical clustering algorithm to group together co-expressed genes and searched the promoters of these genes for consensus binding motifs. They tried to use these clusters to understand the transcriptional mechanisms of cell cycle regulation. Their approach has some drawbacks. First, co-expressed genes are not necessarily co-regulated. Second, even if the genes in a cluster are co-regulated, the relevant TFs still cannot be easily identified by the consensus binding motifs.

**Figure 3:**
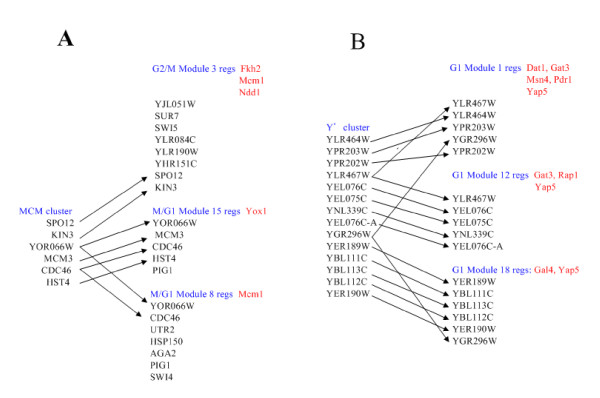
**Refining clusters from Spellman et al.**. (A) Refining the MCM cluster in [7]. The modules identified by MOFA differ not only in the set of TFs regulating the modules, but also in the different cell cycle phases to which they belong, providing a better understanding of how the cell regulates the complex expression program of the yeast cell cycle. Our results confirm previous findings that Mcm1 collaborates with Yox1 to regulate genes in M/G1 (e.g. *YOR066W *and *CDC46*) [30] and collaborates with Ndd1 and Fkh1/Fkh2 to regulate genes in G2/M (e.g. *SPO12 *and *KIN3*) [36]. (B) Refining the Y' cluster in [7]. The Y' cluster contains genes that share DNA sequence similarity and are found in Y' elements, which are located at chromosome ends. Spellman *et al. *[7] did not figure out how these genes are regulated. MOFA reassigns genes in the Y' cluster to three modules and identifies several possible regulators (Dat1, Gal4, Gat3, Hap1, Hap4, Msn4, Pdr1, Rap1 and Yap5), providing information for future experiments.

MOFA can refine clusters in [7] and provide a better understanding of how the cell regulates the complex expression program of the yeast cell cycle. For example, MOFA reassigned genes of the MCM cluster in [7] to several modules. As shown in Figure 3A, these modules differ not only in the set of TFs regulating the modules, but also in the different cell cycle phases to which they belong. Our results confirm previous findings that Mcm1 collaborates with Yox1 to regulate genes in M/G1 (e.g. *YOR066W *and *CDC46*) [30] and collaborates with Ndd1 and Fkh1/Fkh2 to regulate genes in G2/M (e.g. *SPO12 *and *KIN3*) [36]. In addition, MOFA provides regulation information of the Y' cluster in [7]. The Y' cluster contains genes that share DNA sequence similarity and are found in Y' elements, which are located at chromosome ends. Spellman *et al. *[7] did not figure out how these genes are regulated. As shown in Figure 3B, MOFA reassigned genes of the Y' cluster to three modules and identified several possible regulators (Dat1, Gal4, Gat3, Hap1, Hap4, Msn4, Pdr1, Rap1 and Yap5), providing information for future experiments.

## Conclusion

We develop a method, called MOdule Finding Algorithm (MOFA), for reconstructing TRMs of the yeast cell cycle by integrating gene expression data and ChIP-chip data. MOFA identified 87 TRMs, which together contain 336 distinct genes regulated by 40 TFs. From the literature [7,23-25], 139 of the 336 genes and 30 of the 40 TFs are known to be involved in the cell cycle. The biological relevance of each inferred TRM was validated by using existing experimental data, enrichment for genes in the same MIPS functional category [23], known DNA-binding motifs [7], etc. Our analysis shows that different combinations of a fairly small number of TFs are responsible for regulating a large number of genes involved in different cell cycle phases and that there may exist crosstalk between the cell cycle and other cellular processes. Besides, MOFA is capable of finding many novel TF-target gene relationships that could not be identified by using the current ChIP-chip data with the stringent *p*-value cutoff (0.001) or the conventional correlation analysis that only checks the co-expressed relationship. In addition, MOFA can determine the relationships between TFs that regulating the same module and the regulatory roles of these TFs. We found nine activators and six repressors, consistent with the results of previous studies [40-52]. Finally, MOFA refines some clusters proposed by previous studies and provides a better understanding of how the complex expression program of the cell cycle is regulated.

We believe that computational analysis of multiple types of data will be a powerful approach to studying complex biological systems when more and more genomic resources such as genome-wide protein activity data and protein-protein interaction data become available.

## Methods

### Data sets

We use the ChIP-chip data in [19] and the gene expression data (α factor) of the yeast cell cycle in [7]. Spellman *et al. *[7] used Fourier transform to identify 800 putative cell cycle genes (113 genes in M/G1, 300 in G1, 71 in S, 121 in S/G2 and 195 in G2/M). By integrating both types of data, our algorithm tries to reconstruct TRMs for each of the five cell cycle phases.

### Identifying temporal relationships of TF-gene pairs

A cell cycle TF and its binding target are said to have a positively (negatively) temporal relationship if the target gene's expression profile is positively (negatively) correlated with the TF's regulatory profile possibly with time lags. It is known that TF binding affects gene expression in a nonlinear fashion: below some level it has no effect, and above some level the effect may saturate. This type of behavior can be modeled using a sigmoid function. Therefore, we define the regulatory profile of a TF as a sigmoid function like previous studies [55-57].

Temporal Relationship Identification Algorithm (TRIA) is developed to identify TF-gene pairs that have a temporal relationship. Let $\vec{x}$ = (*x*_1_,..., *x*_*N*_) be the gene expression time profile of cell cycle TF *x *and $\vec{y}$ = (*y*_1_,..., *y*_*N*_) be the expression profile of gene *y*. The regulatory profile *RP*($\vec{x}$) = (*f *(*x*_1_),..., *f *(*x*_*N*_)) of TF *x *is defined as a sigmoid function, which is justified by some previous studies [55-57]

$\begin{array}{cc} f(x_{i})=\frac{1}{1+e^{-(x_{i}-\bar{x})/s)}} & i=1,2,\cdots ,N \end{array}$

where $\bar{x}$ is the sample mean and *s *is the sample standard deviation of $\vec{x}$. Compute the correlation between $\vec{y}$ and *RP*($\vec{x}$) with a lag of *k *time points [58,59]:

$\begin{array}{cc} r(k)=\left(\sum_{i=1}^{N-k}\left(y_{i+k}-\bar{y}\right)\left(f(x_{i})-\bar{m}\right)\right)/\left(\sqrt{\sum_{i=1}^{N-k}{\left(y_{i+k}-\bar{y}\right)}^{2}}\cdot \sqrt{\sum_{i=1}^{N-k}{\left(f(x_{i})-\bar{m}\right)}^{2}}\right), & k=0,1,...,L \end{array}$

where $\bar{y}≜\left(\sum_{i=1}^{N-k}y_{i+k}\right)/\left(N-k\right)$, $\bar{m}≜\left(\sum_{i=1}^{N-k}f(x_{i})\right)/\left(N-k\right)$ and *L *is the maximal time lag of the TF's regulatory profile considered. The value of *L *is chosen to make the maximal time lag approximately equal to two consecutive cell cycle phases because Simon *et al. *[17] found cases where a cell cycle TF that expresses in one phase of the cell cycle can regulate genes that function in the next phase.

Then we test the null hypothesis H_0_: *r*(*k*) = 0 and the alternative hypothesis H_1_: *r*(*k*) ≠ 0 by the bootstrap method (see Additional file 3) and get a *p*-value *p*(*k*). The time-lagged correlation (*TlC*) of $\vec{y}$ and *RP*($\vec{x}$) is defined as *r*(*j*) that has the smallest *p*-value (i.e., *TlC*($\vec{y}$, *RP*($\vec{x}$)) = *r*(*j*) if *p*(*j*) ≤ *p*(*k*) ∀*k *≠ *j*). Note that -1 ≤ *TlC*($\vec{y}$, *RP*($\vec{x}$)) ≤ 1. Two possible temporal relationships between $\vec{y}$ and *RP*($\vec{x}$) can be identified by TRIA: $\vec{y}$ and *RP*($\vec{x}$) are (1) positively correlated with a lag of *j *time points if *TlC*($\vec{y}$, *RP*($\vec{x}$)) = *r*(*j*) > 0 &*p*(*j*) ≤ *p*_*Threshold *_and (2) negatively correlated with a lag of *j *time points if *TlC*($\vec{y}$, *RP*($\vec{x}$)) = *r*(*j*) < 0 &*p*_*Threshold*_. The *p*_*Threshold *_is chosen to ensure that we have at most a 5% false discovery rate (FDR) [60].

Two observations motivated us to develop TRIA to detect the temporal relationship between a cell cycle TF and its regulatory targets. First, it has been shown that at least in a few instances, the expression levels of TFs and their target genes were correlated [2,59,61-65]. Although this may not be true for TFs which are mainly regulated at the post-transcriptional level [66,67], it is not a serious problem for many cell cycle TFs whose expression levels significantly varies with times indicating that they are also under highly transcriptional control [39,55,56,59,63,68]. Second, the expression relationship between a TF and its regulatory targets may not be simultaneous but after some time lags [39,53,57,59,63-65,69,70].

TRIA was used to find regulatory targets of cell cycle TFs and its effectiveness was validated by statistically testing for the expression coherence, enrichment of functional groups and conserved binding motifs [71]. We found that when only cell cycle TFs are concerned, TRIA performed better than some pervious algorithms [72,73]. This may result from the fact that the previous algorithms are designed for all kinds of TFs but TRIA is specially designed for cell cycle TFs.

### The MOdule Finding Algorithm (MOFA)

Before describing MOFA, we define some terms.

#### Definition 1

Let *E *= [*e*_*ij*_] be the gene expression matrix whose rows correspond to genes and columns correspond to time points, so that *e*_*ij *_is the expression level of gene *i *at time point *j*.

#### Definition 2

Let *B *= [*b*_*ij*_] be the binding score matrix whose rows correspond to genes and columns correspond to TFs, so that *b*_*ij *_denotes the binding score of TF *j *to bind gene *i*. We set *b*_*ij *_= 4 if the *p*-value for TF *j *to bind gene *i *is ≤ 0.001 in the ChIP-chip data and TF *j *and gene *i *are found to have a temporal relationship; *b*_*ij *_= 3 if *p *≤ 0.001 but no temporal relationship; *b*_*ij *_= 2 if 0.001 <*p *≤ 0.01 and a temporal relationship; *b*_*ij*_= 1 if 0.001 <*p *≤ 0.01 but no temporal relationship; and *b*_*ij *_= 0 if *p *> 0.01.

#### Definition 3

Let *R *be a set of TFs and *C*(*R*, *z*) be the set of target genes to which all the TFs in *R *bind with a score ≥ *z*. In addition, let *SP *be the set of all genes in a specific cell cycle phase (113 genes in M/G1, 300 in G1, 71 in S, 121 in S/G2 and 195 in G2/M).

#### Definition 4

The expression coherence score (*EC*(*A*)) for a set *A *is defined as the fraction of gene-gene pairs in *A *whose gene expression profiles are positively correlated possibly with time lags: 0 ≤ *EC*(*A*) ≤ 1. Note that the higher the *EC*(*A*) is, the more plausible the genes in *A *are co-regulated.

#### Remark

The *EC*(*A*) is a generalization of the expression correlation score used in [13,20]. Compared to theirs, our measure can in addition find co-regulated genes whose gene expression profiles are positively correlated with time lags. As shown in [53,54], co-regulated genes are not necessarily co-expressed. Since each gene may have a different response time to the same transcriptional regulatory mechanism in transcribing DNA to RNA, the RNA profiles of co-regulated genes may not be co-expressed but rather postivley correlated with time lags.

**Figure 4:**
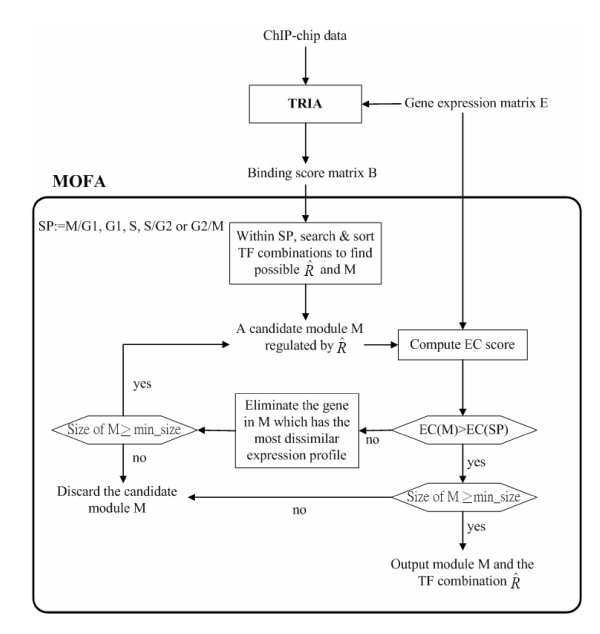
Flowchart of MOFA.

**Figure 5:**
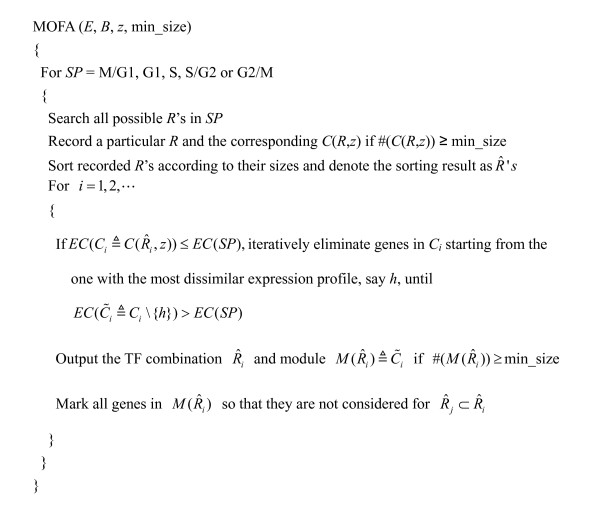
**The pseudocode of MOFA**. In this study, we set the binding score *z *= 2. This means that a TF is regarded as binding to a gene if (1) the *p*-value for the TF to bind the gene is ≤ 0.001 in the ChIP-chip data or (2) 0.001<*p *≤ 0.01 and the TF-gene pair have a temporal relationship. Moreover, we require that the number of genes in a module must be ≥ 5. This value is the same as that in GRAM algorithm [21] for comparison purpose.

MOFA performs in two steps (see Figure 4). First, for a specific cell cycle phase (M/G1, G1, S, S/G2 or G2/M), it exhaustively searches all possible *R *'*s *in order to find *C*(*R*, *z*)'*s*. A particular *R *and the corresponding *C*(*R*, *z*) are recorded if *C*(*R*, *z*) contains more than a certain number of genes. MOFA then sorts the recorded *R *'*s *according to their sizes, denoting the sorting result as $\hat{R}$'*s*, so that the first $\hat{R}$ is the one with the largest number of members. Second, if *EC*(*C*($\hat{R}$, *z*)) ≤ *EC*(*SP*), MOFA iteratively eliminates genes of the set *C*($\hat{R}$, *z*) starting from the one with the most dissimilar expression profile until *EC*($\tilde{C}$($\hat{R}$, *z*)) > *EC*(*SP*), where $\tilde{C}$($\hat{R}$, *z*) is the set of the remaining genes and *SP *is the set of all genes in a specific cell cycle phase. That is, MOFA tries to identify a subset of co-regulated genes whose gene expression profiles are highly positively correlated possibly with time lags compared to that of the set of all genes in the specific cell cycle phase. Finally, MOFA outputs a module *M *($\hat{R}$) ≜ $\tilde{C}$($\hat{R}$, *z*) if $\tilde{C}$($\hat{R}$, *z*) contains more than a certain number of genes, say five. The above procedure goes over all $\hat{R}$'*s *in the specific cell cycle phase. We provide the pseudocode of MOFA in Figure 5.

## Authors' contributions

WSW developed the algorithm, performed the simulation and wrote the manuscript. WHL and BSC gave the research topic, provided essential guidance and revised the manuscript. All authors read and approved the final manuscript.

## Supplementary Material

Additional file 1 Supplementary Table 1 Click here for file

Additional file 2 Supplementary Table 2 Click here for file

Additional file 3 Supplementary Table 3 Click here for file

Additional file 4 Supplementary Figure 1 Click here for file
