# Supplementary material for: Computational reconstruction of transcriptional regulatory modules of the yeast cell cycle
Source: BMC Bioinformatics. 2006 Sep 29;7:421. doi: 10.1186/1471-2105-7-421 (PMC1637117; doi:10.1186/1471-2105-7-421)
Supplement: Additional file 2 — Supplementary Table 2 [file 1471-2105-7-421-S2.pdf]

Supplementary Table 2 The largest over-represented MIPS functional category of each module with adjusted *P*-value ≤ 0.05 (after Bonferroni correction for multiple tests) using cumulative hypergeometric distribution.

M/G1 phase

Module 1

FUNCTIONAL CATEGORY	YOUR GENE MATCHES	GENOME MATCHES	P-VALUE
**10.01.05.03.03 somatic / mitotic recombination**	**3 entries (60%) (ydr545w ylr466w ypl283c) *annotated-FunCats***	**21 cat entries (0.31%) *annotated-FunCats***	**2.74e-07**.

Module 2

FUNCTIONAL CATEGORY	YOUR GENE MATCHES	GENOME MATCHES	P-VALUE

Module 3

FUNCTIONAL CATEGORY	YOUR GENE MATCHES	GENOME MATCHES	P-VALUE
**34.11.03.07 pheromone response, mating-type determination, sex-specific proteins**	**4 entries (80%) (ycl027w ycl055w yhr005c ylr452c) *annotated-FunCats***	**189 cat entries (2.85%) *annotated-FunCats***	**3.14e-06**.

Module 4

FUNCTIONAL CATEGORY	YOUR GENE MATCHES	GENOME MATCHES	P-VALUE

Module 5

FUNCTIONAL CATEGORY	YOUR GENE MATCHES	GENOME MATCHES	P-VALUE
01.06.01 lipid, fatty acid and isoprenoid biosynthesis	3 entries (37.5%) (ykl182w ymr246w yor317w) *annotated-FunCats*	171 cat entries (2.58%) *annotated-FunCats*	8.61e-04.

Module 6

FUNCTIONAL CATEGORY	YOUR GENE MATCHES	GENOME MATCHES	P-VALUE
01.06.01 lipid, fatty acid and isoprenoid biosynthesis	3 entries (33.3%) (ygl055w ykl182w yor317w) *annotated-FunCats*	171 cat entries (2.58%) *annotated-FunCats*	1.27e-03.

Module 7

FUNCTIONAL CATEGORY	YOUR GENE MATCHES	GENOME MATCHES	P-VALUE
10.03.01 mitotic cell cycle and cell cycle control	3 entries (60%) (yer111c yjl194w ynl078w) *annotated-FunCats*	442 cat entries (6.67%) *annotated-FunCats*	2.67e-03.

Module 8

FUNCTIONAL CATEGORY	YOUR GENE MATCHES	GENOME MATCHES	P-VALUE

Module 9

FUNCTIONAL	YOUR GENE	GENOME	P-VALUE

CATEGORY	MATCHES	MATCHES	

Module 10

FUNCTIONAL CATEGORY	YOUR GENE MATCHES	GENOME MATCHES	P-VALUE

Module 11

FUNCTIONAL CATEGORY	YOUR GENE MATCHES	GENOME MATCHES	P-VALUE

Module 12

FUNCTIONAL CATEGORY	YOUR GENE MATCHES	GENOME MATCHES	P-VALUE
01.06.01.05 fatty acid biosynthesis	2 entries (40%) (ygl055w yjl196c) *annotated-FunCats*	14 cat entries (0.21%) *annotated-FunCats*	4.14e-05.

Module 13

FUNCTIONAL CATEGORY	YOUR GENE MATCHES	GENOME MATCHES	P-VALUE

Module 14

FUNCTIONAL CATEGORY	YOUR GENE MATCHES	GENOME MATCHES	P-VALUE

Module 15

FUNCTIONAL	YOUR GENE	GENOME MATCHES	P-VALUE

CATEGORY	MATCHES		
10.01 DNA processing	3 entries (60%) (ydr191w yel032w ylr274w) *annotated-FunCats*	512 cat entries (7.73%) *annotated-FunCats*	4.08e-03.

G1 phase

Module 1

FUNCTIONAL CATEGORY	YOUR GENE MATCHES	GENOME MATCHES	P-VALUE
10.01.05.03.03 somatic / mitotic recombination	2 entries (40%) (ygr296w ylr467w) *annotated-FunCats*	21 cat entries (0.31%) *annotated-FunCats*	9.52e-05.

Module 2

FUNCTIONAL CATEGORY	YOUR GENE MATCHES	GENOME MATCHES	P-VALUE

Module 3

FUNCTIONAL CATEGORY	YOUR GENE MATCHES	GENOME MATCHES	P-VALUE

Module 4

FUNCTIONAL CATEGORY	YOUR GENE MATCHES	GENOME MATCHES	P-VALUE

Module 5

FUNCTIONAL CATEGORY	YOUR GENE MATCHES	GENOME MATCHES	P-VALUE
**10.01.05.03.03 somatic / mitotic recombination**	**3 entries (60%) (ygr296w ylr467w ynl339c) *annotated-FunCats***	**21 cat entries (0.31%) *annotated-FunCats***	**2.74e-07**.

Module 6

FUNCTIONAL CATEGORY	YOUR GENE MATCHES	GENOME MATCHES	P-VALUE

Module 7

FUNCTIONAL CATEGORY	YOUR GENE MATCHES	GENOME MATCHES	P-VALUE
**10.01.05.03.03 somatic / mitotic recombination**	**4 entries (80%) (yer190w ygr296w ylr467w ynl339c) *annotated-FunCats***	**21 cat entries (0.31%) *annotated-FunCats***	**3.73e-10**.

Module 8

FUNCTIONAL CATEGORY	YOUR GENE MATCHES	GENOME MATCHES	P-VALUE
**02.19 metabolism of energy reserves (e.g. glycogen, trehalose)**	**2 entries (40%) (ylr300w yml100w) *annotated-FunCats***	**56 cat entries (0.84%) *annotated-FunCats***	**6.91e-04**.

Module 9

FUNCTIONAL	YOUR GENE	GENOME	P-VALUE

CATEGORY	MATCHES	MATCHES	

Module 10

FUNCTIONAL CATEGORY	YOUR GENE MATCHES	GENOME MATCHES	P-VALUE

Module 11

FUNCTIONAL CATEGORY	YOUR GENE MATCHES	GENOME MATCHES	P-VALUE
10.01.03.01 DNA topology	2 entries (40%) (ybl113c yml133c) *annotated-FunCats*	54 cat entries (0.81%) *annotated-FunCats*	6.43e-04.

Module 12

FUNCTIONAL CATEGORY	YOUR GENE MATCHES	GENOME MATCHES	P-VALUE
10.01.05.03.03 somatic / mitotic recombination	2 entries (40%) (ylr467w ynl339c) *annotated-FunCats*	21 cat entries (0.31%) *annotated-FunCats*	9.52e-05.

Module 13

FUNCTIONAL CATEGORY	YOUR GENE MATCHES	GENOME MATCHES	P-VALUE
10 CELL CYCLE AND DNA PROCESSING	7 entries (63.6%) (ydl003w ydl227c ydr507c yer070w yjl187c yml027w ypr120c) *annotated-FunCats*	1000 cat entries (15.1%) *annotated-FunCats*	3.31e-04.

Module 14

FUNCTIONAL CATEGORY	YOUR GENE MATCHES	GENOME MATCHES	P-VALUE

Module 15

FUNCTIONAL CATEGORY	YOUR GENE MATCHES	GENOME MATCHES	P-VALUE
01.05.01 C-compound and carbohydrate utilization	3 entries (60%) (ygl028c yhr143w yml100w) *annotated-FunCats*	389 cat entries (5.87%) *annotated-FunCats*	1.84e-03.

Module 16

FUNCTIONAL CATEGORY	YOUR GENE MATCHES	GENOME MATCHES	P-VALUE

Module 17

FUNCTIONAL CATEGORY	YOUR GENE MATCHES	GENOME MATCHES	P-VALUE

Module 18

FUNCTIONAL CATEGORY	YOUR GENE MATCHES	GENOME MATCHES	P-VALUE
10.01.03.01 DNA topology	3 entries (50%) (ybl113c yer190w ygr296w) *annotated-FunCats*	54 cat entries (0.81%) *annotated-FunCats*	1.01e-05.

Module 19

FUNCTIONAL CATEGORY	YOUR GENE MATCHES	GENOME MATCHES	P-VALUE
10 CELL CYCLE AND DNA PROCESSING	6 entries (75%) (ydl101c yil026c ykl113c ynl273w yor074c ypr075c) *annotated-FunCats*	1000 cat entries (15.1%) *annotated-FunCats*	2.49e-04.

Module 20

FUNCTIONAL CATEGORY	YOUR GENE MATCHES	GENOME MATCHES	P-VALUE
01.05.01 C-compound and carbohydrate utilization	3 entries (60%) (yer001w ylr300w ylr342w) *annotated-FunCats*	389 cat entries (5.87%) *annotated-FunCats*	1.84e-03.

Module 21

FUNCTIONAL CATEGORY	YOUR GENE MATCHES	GENOME MATCHES	P-VALUE

Module 22

FUNCTIONAL CATEGORY	YOUR GENE MATCHES	GENOME MATCHES	P-VALUE

Module 23

FUNCTIONAL CATEGORY	YOUR GENE MATCHES	GENOME MATCHES	P-VALUE

Module 24

FUNCTIONAL CATEGORY	YOUR GENE MATCHES	GENOME MATCHES	P-VALUE

Module 25

FUNCTIONAL CATEGORY	YOUR GENE MATCHES	GENOME MATCHES	P-VALUE

Module 26

FUNCTIONAL CATEGORY	YOUR GENE MATCHES	GENOME MATCHES	P-VALUE
**10.03.01 mitotic cell cycle and cell cycle control**	**3 entries (60%) (ygr188c ymr078c ypr075c) *annotated-FunCats***	**442 cat entries (6.67%) *annotated-FunCats***	**2.67e-03**.

Module 27

FUNCTIONAL CATEGORY	YOUR GENE MATCHES	GENOME MATCHES	P-VALUE
**10 CELL CYCLE AND DNA PROCESSING**	**7 entries (53.8%) (yar007c ycl061c ydr097c yel077c ygr188c yhr143w yjl074c) *annotated-FunCats***	**1000 cat entries (15.1%) *annotated-FunCats***	**1.30e-03**.

Module 28

FUNCTIONAL CATEGORY	YOUR GENE MATCHES	GENOME MATCHES	P-VALUE

**10.01.03.01 DNA topology**	3 entries (60%) (yel077c yer190w yil177c) *annotated-FunCats*	54 cat entries (0.81%) *annotated-FunCats*	5.07e-06.

Module 29

FUNCTIONAL CATEGORY	YOUR GENE MATCHES	GENOME MATCHES	P-VALUE
**02.19 metabolism of energy reserves (e.g. glycogen, trehalose)**	2 entries (40%) (yml100w ypr160w) *annotated-FunCats*	56 cat entries (0.84%) *annotated-FunCats*	6.91e-04.

Module 30

FUNCTIONAL CATEGORY	YOUR GENE MATCHES	GENOME MATCHES	P-VALUE
**10.01.03 DNA synthesis and replication**	3 entries (37.5%) (yer070w ynl262w yor074c) *annotated-FunCats*	179 cat entries (2.70%) *annotated-FunCats*	9.84e-04.

Module 31

FUNCTIONAL CATEGORY	YOUR GENE MATCHES	GENOME MATCHES	P-VALUE
**01.05.01.01.02 polysaccharide degradation**	2 entries (40%) (ymr305c ypr160w) *annotated-FunCats*	19 cat entries (0.28%) *annotated-FunCats*	7.76e-05.

Module 32

FUNCTIONAL	YOUR GENE	GENOME MATCHES	P-VALUE

CATEGORY	MATCHES		
10.01.03.01 DNA topology	3 entries (42.8%) (ylr467w yml133c ynl339c) annotated-FunCats	54 cat entries (0.81%) annotated-FunCats	1.75e-05.

Module 33

FUNCTIONAL CATEGORY	YOUR GENE MATCHES	GENOME MATCHES	P-VALUE

Module 34

FUNCTIONAL CATEGORY	YOUR GENE MATCHES	GENOME MATCHES	P-VALUE

Module 35

FUNCTIONAL CATEGORY	YOUR GENE MATCHES	GENOME MATCHES	P-VALUE
42 BIOGENESIS OF CELLULAR COMPONENTS	5 entries (62.5%) (yer190w ygr014w ygr221c yjl187c ylr467w) annotated-FunCats	854 cat entries (12.8%) annotated-FunCats	1.41e-03.

Module 36

FUNCTIONAL CATEGORY	YOUR GENE MATCHES	GENOME MATCHES	P-VALUE
10.03 cell cycle	4 entries (66.6%) (ycl024w yhr153c ylr286c ymr076c)	648 cat entries (9.78%) annotated-FunCats	1.16e-03.

	annotated-FunCats		

S phase

Module 1

FUNCTIONAL CATEGORY	YOUR GENE MATCHES	GENOME MATCHES	P-VALUE
16.03.01 DNA binding	6 entries (100%) (ybr009c ybr010w ydr224c ydr225w ynl030w ynl031c) *annotated-FunCats*	159 cat entries (2.40%) *annotated-FunCats*	1.75e-10

Module 2

FUNCTIONAL CATEGORY	YOUR GENE MATCHES	GENOME MATCHES	P-VALUE
11.02.03.04 transcriptional control	3 entries (60%) (ybl002w ybl003c ydr451c) *annotated-FunCats*	492 cat entries (7.42%) *annotated-FunCats*	3.64e-03.

Module 3

FUNCTIONAL CATEGORY	YOUR GENE MATCHES	GENOME MATCHES	P-VALUE
11.02.03.04 transcriptional control	4 entries (57.1%) (ydr224c ydr225w ydr451c ypl127c) *annotated-FunCats*	492 cat entries (7.42%) *annotated-FunCats*	8.79e-04.

Module 4

FUNCTIONAL CATEGORY	YOUR GENE MATCHES	GENOME MATCHES	P-VALUE

Module 5

FUNCTIONAL CATEGORY	YOUR GENE MATCHES	GENOME MATCHES	P-VALUE
**11.02.03.04 transcriptional control**	**3 entries (60%) (ybr009c ydr451c ypr034w) *annotated-FunCats***	**492 cat entries (7.42%) *annotated-FunCats***	**3.64e-03**.

Module 6

FUNCTIONAL CATEGORY	YOUR GENE MATCHES	GENOME MATCHES	P-VALUE
**11.02.03.04 transcriptional control**	**3 entries (60%) (ybr009c ybr010w ydr451c) *annotated-FunCats***	**492 cat entries (7.42%) *annotated-FunCats***	**3.64e-03**.

S/G2 phase

Module 1

FUNCTIONAL CATEGORY	YOUR GENE MATCHES	GENOME MATCHES	P-VALUE

Module 2

FUNCTIONAL CATEGORY	YOUR GENE MATCHES	GENOME MATCHES	P-VALUE

Module 3

FUNCTIONAL CATEGORY	YOUR GENE MATCHES	GENOME MATCHES	P-VALUE

Module 4

FUNCTIONAL CATEGORY	YOUR GENE MATCHES	GENOME MATCHES	P-VALUE
10.03 cell cycle	5 entries (50%) (ydr130c yil135c yir010w ykl048c ymr198w) *annotated-FunCats*	648 cat entries (9.78%) *annotated-FunCats*	1.46e-03.

Module 5

FUNCTIONAL CATEGORY	YOUR GENE MATCHES	GENOME MATCHES	P-VALUE

Module 6

FUNCTIONAL CATEGORY	YOUR GENE MATCHES	GENOME MATCHES	P-VALUE
01.01.03.03.02 degradation of proline	1 entries (20%) (ylr142w) *annotated-FunCats*	4 cat entries (0.06%) *annotated-FunCats*	3.02e-03.

Module 7

FUNCTIONAL CATEGORY	YOUR GENE MATCHES	GENOME MATCHES	P-VALUE
11.02.03.04 transcriptional control	4 entries (80%) (yhr178w yml065w yor337w ypl133c) *annotated-FunCats*	492 cat entries (7.42%) *annotated-FunCats*	1.42e-04.

Module 8

FUNCTIONAL CATEGORY	YOUR GENE MATCHES	GENOME MATCHES	P-VALUE

G2/M phase

Module 1

FUNCTIONAL CATEGORY	YOUR GENE MATCHES	GENOME MATCHES	P-VALUE
10.03.01.01 mitotic cell cycle	3 entries (42.8%) (ygl116w ylr131c ypr119w) *annotated-FunCats*	164 cat entries (2.47%) *annotated-FunCats*	4.85e-04.

Module 2

FUNCTIONAL CATEGORY	YOUR GENE MATCHES	GENOME MATCHES	P-VALUE

Module 3

FUNCTIONAL CATEGORY	YOUR GENE MATCHES	GENOME MATCHES	P-VALUE

**43 CELL TYPE DIFFERENTIATION**	**4 entries (50%) (yar018c yhr152w ylr084c yml052w) *annotated-FunCats***	**449 cat entries (6.78%) *annotated-FunCats***	**1.17e-03**.

Module 4

FUNCTIONAL CATEGORY	YOUR GENE MATCHES	GENOME MATCHES	P-VALUE
**10.03.01 mitotic cell cycle and cell cycle control**	**3 entries (60%) (yhr152w ykr042w ypr119w) *annotated-FunCats***	**442 cat entries (6.67%) *annotated-FunCats***	**2.67e-03**.

Module 5

FUNCTIONAL CATEGORY	YOUR GENE MATCHES	GENOME MATCHES	P-VALUE
**10.03 cell cycle**	**4 entries (66.6%) (yar018c ygr092w yhr152w ykr042w) *annotated-FunCats***	**648 cat entries (9.78%) *annotated-FunCats***	**1.16e-03**.

Module 6

FUNCTIONAL CATEGORY	YOUR GENE MATCHES	GENOME MATCHES	P-VALUE

Module 7

FUNCTIONAL CATEGORY	YOUR GENE MATCHES	GENOME MATCHES	P-VALUE

Module 8

FUNCTIONAL CATEGORY	YOUR GENE MATCHES	GENOME MATCHES	P-VALUE

Module 9

FUNCTIONAL CATEGORY	YOUR GENE MATCHES	GENOME MATCHES	P-VALUE
**02 ENERGY**	**4 entries (44.4%) (ygr240c ynl037c ypl036w ypr128c) *annotated-FunCats***	**365 cat entries (5.51%) *annotated-FunCats***	**9.17e-04**.

Module 10

FUNCTIONAL CATEGORY	YOUR GENE MATCHES	GENOME MATCHES	P-VALUE

Module 11

FUNCTIONAL CATEGORY	YOUR GENE MATCHES	GENOME MATCHES	P-VALUE
**02.13.01 anaerobic respiration**	**1 entries (14.2%) (ybl030c) *annotated-FunCats***	**4 cat entries (0.06%) *annotated-FunCats***	**4.22e-03**.

Module 12

FUNCTIONAL CATEGORY	YOUR GENE MATCHES	GENOME MATCHES	P-VALUE

Module 13

FUNCTIONAL CATEGORY	YOUR GENE MATCHES	GENOME MATCHES	P-VALUE
**10.03.01.01.11 mitosis**	**2 entries (40%) (yml064c ynl172w) *annotated-FunCats***	**51 cat entries (0.77%) *annotated-FunCats***	**5.73e-04**.

Module 14

FUNCTIONAL CATEGORY	YOUR GENE MATCHES	GENOME MATCHES	P-VALUE

Module 15

FUNCTIONAL CATEGORY	YOUR GENE MATCHES	GENOME MATCHES	P-VALUE

Module 16

FUNCTIONAL CATEGORY	YOUR GENE MATCHES	GENOME MATCHES	P-VALUE

Module 17

FUNCTIONAL CATEGORY	YOUR GENE MATCHES	GENOME MATCHES	P-VALUE
**02.13.01 anaerobic respiration**	**1 entries (20%) (ybl030c) *annotated-FunCats***	**4 cat entries (0.06%) *annotated-FunCats***	**3.02e-03**.

Module 18

FUNCTIONAL CATEGORY	YOUR GENE MATCHES	GENOME MATCHES	P-VALUE

Module 19

FUNCTIONAL CATEGORY	YOUR GENE MATCHES	GENOME MATCHES	P-VALUE
20.01 transported compounds (substrates)	6 entries (75%) (ybr069c yer145c ygl162w yml116w yor049c yor153w) *annotated-FunCats*	585 cat entries (8.83%) *annotated-FunCats*	1.11e-05.

Module 20

FUNCTIONAL CATEGORY	YOUR GENE MATCHES	GENOME MATCHES	P-VALUE

Module 21

FUNCTIONAL CATEGORY	YOUR GENE MATCHES	GENOME MATCHES	P-VALUE

Module 22

FUNCTIONAL CATEGORY	YOUR GENE MATCHES	GENOME MATCHES	P-VALUE
