# Supplementary material for: Computational reconstruction of transcriptional regulatory modules of the yeast cell cycle
Source: BMC Bioinformatics. 2006 Sep 29;7:421. doi: 10.1186/1471-2105-7-421 (PMC1637117; doi:10.1186/1471-2105-7-421)
Supplement: Additional file 3 — Supplementary Table 3 [file 1471-2105-7-421-S3.pdf]

The bootstrap method for testing the statistical significance of the difference between *r*(*k*) and 0.

We observed N-k pairs of observations, $Z=\{\mathbf{z}_i$: i=1, $\cdots$, N-k and $\mathbf{z}_i=(f(x_i),y_{i+k})\}$. The correlation coefficient from the sample is calculated and denoted as

$$r(k)=\left(\sum_{i=1}^{N-k}(y_{i+k}-\bar{y})(f(x_i)-\bar{m})\right)\Bigg/\left(\sqrt{\sum_{i=1}^{N-k}(y_{i+k}-\bar{y})^2}\cdot\sqrt{\sum_{i=1}^{N-k}(f(x_i)-\bar{m})^2}\right),\ k=0,1,2,\cdots$$

where $\bar{y}\triangleq\left(\sum_{i=1}^{N-k}y_{i+k}\right)\Big/(N-k)$, $\bar{m}\triangleq\left(\sum_{i=1}^{N-k}f(x_i)\right)\Big/(N-k)$ and $-1\le r(k)\le 1$. It is aimed to use these observations to test if $r(k)$ is different from 0 significantly. Suppose the null hypothesis is H_0: $r(k)=0$ and the alternative hypothesis is H_1: $r(k)\neq 0$. We will apply the bootstrap method to perform this hypothesis testing based on the observations. Keeping the pair relationship of these N-k pairs to maintain the dependence between $(f(x_i),y_{i+k})$, $\mathbf{z}_i$ are sampled with replacement N-k times to form a bootstrap sample, $Z^*=\{\mathbf{z}_i^*$: i=1, $\cdots$, N-k and $\mathbf{z}_i^*$ belongs to $Z\}$. The correlation coefficient from the bootstrap sample Z^* is computed and denoted as $r^*(k)$, $-1\le r^*(k)\le 1$.

Repeat the resampling procedure B times, we will observed $r_1^*(k), r_2^*(k), ..., r_B^*(k)$. These bootstrap correlation coefficients are sorted to be $-1\le r_{(1)}^*(k)\le r_{(2)}^*(k)\le ...\le r_{(B)}^*(k)\le 1$. Then, the (1-α) two-sided percentile interval is given by $[r^*_{(B\times\alpha/2)}(k),\ r^*_{(B\times(1-\alpha/2))}(k)]$ in this case [1]. If this percentile interval does not contain 0, then the null hypothesis is rejected at the significance level of α. Otherwise, the data fail to reject the null hypothesis at the significance level of α. Since the p-value is the smallest value of α for which the null hypothesis will be rejected based on the observation, the p-value for this test is estimated by the following:

$$\hat{p}(k)=2\times\min\{\hat{p}_+(k),1-\hat{p}_+(k)\},\ \text{where}\ \hat{p}_+(k)=\sum_{i=1}^{B}\mathrm{I}\{r_i^*(k)\ge 0\}/B,$$

where $\mathbf{I}\{\cdot\}$ is the indicator function whose value is one when the event is true and zero otherwise.

[1] Efron B, Tibshirani RJ: *An introduction to the Bootstrap.* London: Chapman Hall; 1993.
