# Supplementary material for: Computational reconstruction of transcriptional regulatory modules of the yeast cell cycle
Source: BMC Bioinformatics. 2006 Sep 29;7:421. doi: 10.1186/1471-2105-7-421 (PMC1637117; doi:10.1186/1471-2105-7-421)
Supplement: Additional file 4 — Supplementary Figure 1 [file 1471-2105-7-421-S4.pdf]

A

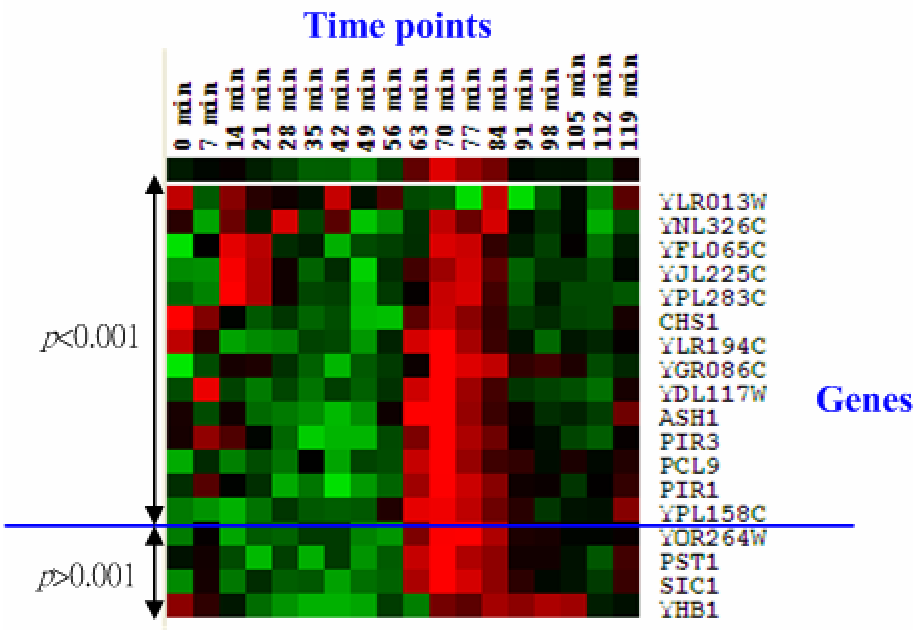
Time points
0 min
7 min
14 min
21 min
28 min
35 min
42 min
49 min
56 min
63 min
70 min
77 min
84 min
91 min
98 min
105 min
112 min
119 min
p<0.001
p>0.001
YLR013W
YNL326C
YFL065C
YJL225C
YPL283C
CHS1
YLR194C
YGR086C
YDL117W
ASH1
PIR3
PCL9
PIR1
YPL158C
YOR264W
PST1
SIC1
YHB1
Genes

B

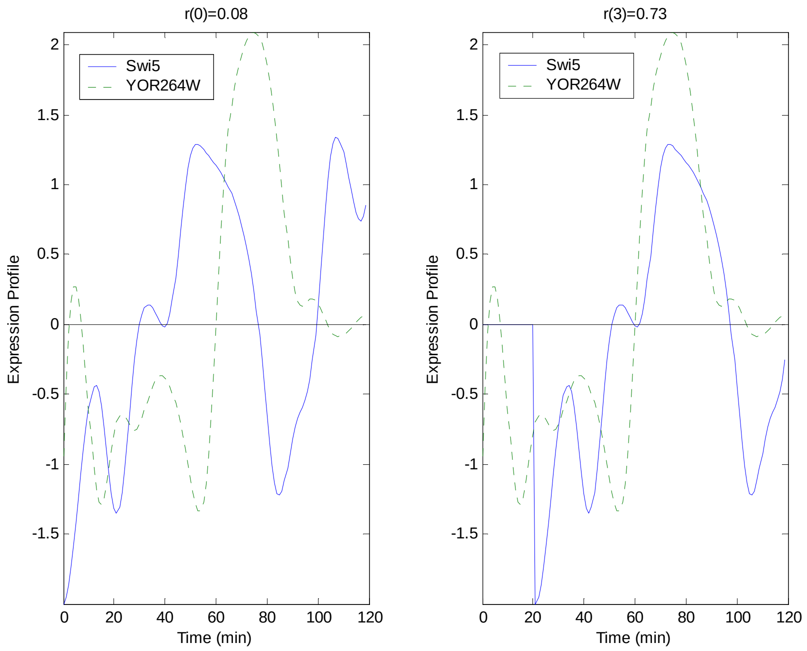
r(0)=0.08
r(3)=0.73
Swi5
YOR264W
Expression Profile
Time (min)

C

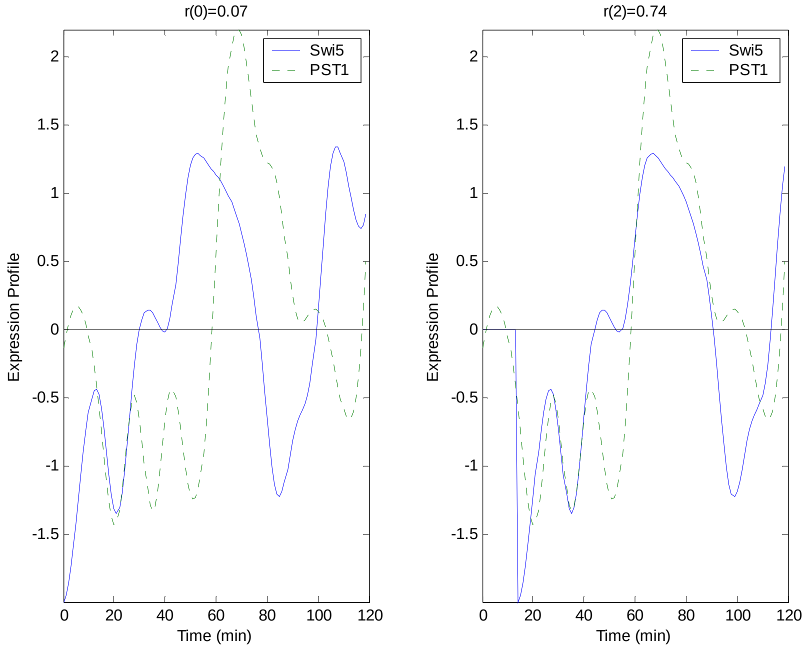
r(0)=0.07
r(2)=0.74
Swi5
PST1
Expression Profile
Time (min)

D

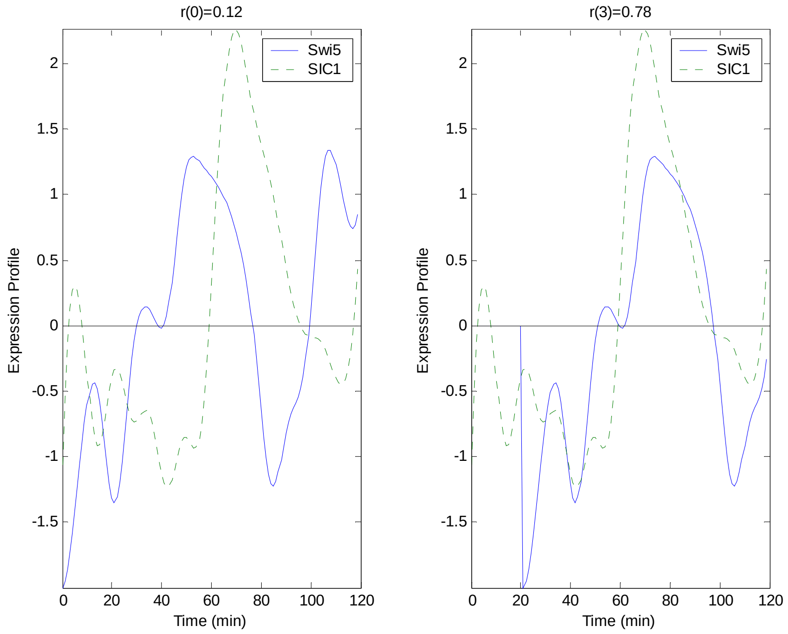
r(0)=0.12
r(3)=0.78
Swi5
SIC1
Expression Profile
Time (min)

E

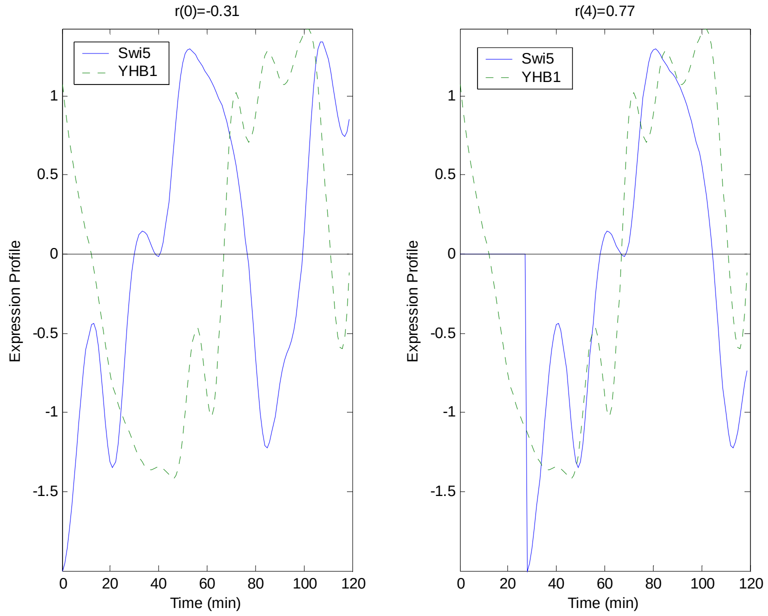

Supplementary Figure 1 MOFA can reduce false negatives in determining binding events in the ChIP-chip data. (A) Gene expression patterns of the 18 target genes of the {Swi5} module in M/G1. **(B)** TF-gene pair Swi5-*YOR264W* with binding *p*-value=0.0053>0.001. **(C)** TF-gene pair Swi5-*PST1* with binding *p*-value=0.0085>0.001. **(D)** TF-gene pair Swi5-*SIC1* with binding *p*-value=0.0017>0.001. **(E)** TF-gene pair Swi5-*YHB1* with binding *p*-value=0.007>0.001. These four TF-gene pairs all have positively time-shifted correlated relationships. Note that these four TF-gene pairs identified by MOFA could not be identified by using the current ChIP-chip data with the stringent *p*-value cutoff (0.001) or the conventional correlation analysis that can only check co-expressed relationship.
